# How to benchmark medical AI agents

**DOI:** 10.1371/journal.pmed.1005170

**Published:** 2026-07-09

**Authors:** Silas Ruhrberg Estévez, Dyke Ferber, Mihaela van der Schaar, Jakob Nikolas Kather

**Affiliations:** 1 Else Kroener Fresenius Center for Digital Health, Faculty of Medicine and University Hospital Carl Gustav Carus, TUD Dresden University of Technology, Dresden, Germany; 2 Cambridge Centre for AI in Medicine, University of Cambridge, Cambridge, United Kingdom; 3 Department of Applied Mathematics and Theoretical Physics, University of Cambridge, Cambridge, United Kingdom; 4 Department of Medicine I, Faculty of Medicine and University Hospital Carl Gustav Carus, TUD Dresden University of Technology, Dresden, Germany; 5 Medical Oncology, National Center for Tumor Diseases (NCT), University Hospital Heidelberg, Heidelberg, Germany; 6 Pathology & Data Analytics, Leeds Institute of Medical Research at St James’s, University of Leeds, Leeds, United Kingdom

## Abstract

In this Perspective article, Silas Ruhrberg Estévez and colleagues discuss why, as medical AI research shifts toward multimodal large language model-based agents for complex clinical workflows, benchmarks that assess clinical reasoning, process safety, and resource stewardship—rather than final outputs alone—are required.

## Teaser

Medical artificial intelligence research is shifting from single-task models toward multimodal large language model-based agents for complex clinical workflows, requiring benchmarks that assess clinical reasoning, process safety, and resource stewardship rather than final outputs alone.

## Introduction

Before deployment in high-stakes settings, artificial intelligence (AI) systems must be shown to be accurate, safe, generalizable, and useful for their intended tasks [1]. In machine learning, standardized benchmarks allow algorithms to be compared under shared conditions. They do more than measure performance: They define the problem, determine what counts as success, and shape scientific progress. ImageNet illustrates this effect by providing a large-scale, standardized task that helped drive modern computer vision [2]. Medical AI has followed the same benchmarking logic; radiology datasets have enabled systematic comparison of imaging models [3], while question-answering benchmarks such as MedQA [4] assess clinical reasoning. These benchmarks share a simple evaluation structure: a fixed input, a single response, and a reference answer against which predictions are scored. This structure has been effective in measuring and accelerating progress on bounded clinical tasks, from image interpretation and histopathology classification to knowledge-based medical licensing examinations, with some systems reaching expert-level performance under controlled benchmark conditions [5,6].

**Fig 1:**
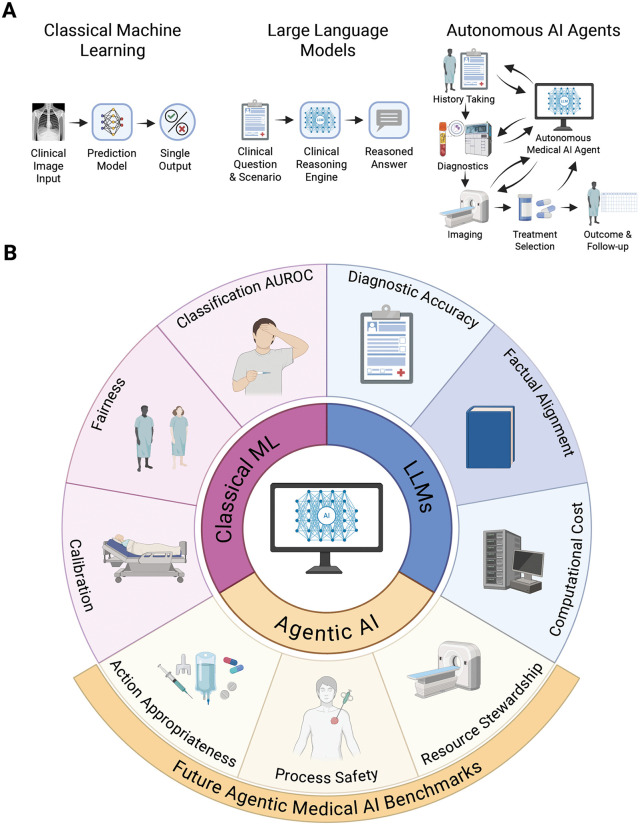
Evaluation settings for medical AI models. **(A)** Schematic contrast between classical machine-learning models, LLM-based clinical question answering, and autonomous medical AI agents embedded in clinical workflows. **(B)** Overview of benchmark domains across model generations, highlighting the additional evaluation dimensions required for agentic systems: action appropriateness, process safety, and resource stewardship. Figure was created in BioRender. Ruhrberg Estévez, S. (2026) https://BioRender.com/s6mb56s.

As illustrated in Fig 1A, classical machine-learning models typically map fixed clinical inputs to prediction outputs, while large language models (LLMs) generate text responses to clinical questions. Medical AI agents differ because they operate within a clinical workflow: they actively gather information, use tools, and adapt subsequent decisions as new information becomes available. Final-output matching is therefore insufficient for agentic workflows, because clinical care often allows multiple safe trajectories. Competence depends on how information is gathered, actions are sequenced, and decisions adapt over time. Emerging benchmarks have begun to reflect this through simulated patient encounters and iterative diagnostic tasks [7]. In these settings, performance is more variable, suggesting that earlier benchmarks primarily rewarded static answers rather than longitudinal decision-making [6,8].

## Benchmarking sequential decision-making

Similar evaluation paradigms already exist outside medicine. In interactive environments such as video games, evaluation extends beyond recalling facts about the environment and instead assesses whether agents can observe the current state, select actions, adapt to feedback, and pursue long-horizon objectives [9]. These environments distinguish an agent’s strategic choices from the mechanics of action execution. For example, an agent playing a video game such as Pokémon must decide when to explore, collect characters, interact with non-player characters, or enter battles; success depends on how these decisions are sequenced as the game state evolves.

The same distinction applies to medical agents operating within an electronic health record. Clinical decision-making is sequential, multimodal, and constrained by cost, time, resource availability, and patient safety. The agent must decide whether to ask further history questions, perform examination steps, order laboratory tests or imaging, request specialist input, consult guidelines, or initiate treatment. Existing benchmarks remain valuable for evaluating these component capabilities. In agentic systems, however, the same models increasingly function as tools within a broader decision-making workflow rather than as standalone predictors [5,10].

Early clinical agent benchmarks illustrate why workflow-level evaluation is necessary. Multi-step procedures create failure modes invisible to endpoint scoring, including shortcut learning, premature closure, unnecessary tool use, and clinically implausible paths to otherwise correct answers [8,11]. Recent evaluations of LLM-based clinical agents show that adding tools does not automatically produce reliable clinical behavior, with only modest gains over baseline models, as well as persistently low performance in several diagnostic and multimodal settings, and substantially increased resource use [12]. Process-aware evaluation can address these limitations by measuring deviation from reference workups, completion of safety checks, guideline adherence, and proportionality of resource use. Such benchmarks would direct model development toward clinically meaningful behavior and provide developers, clinicians, and regulators with more interpretable evidence for assessing readiness before deployment.

## Benchmarking medical AI agents

In an emergency department workup, a patient presenting with abdominal pain is assessed iteratively: history-taking informs laboratory testing, which guides imaging and treatment decisions. Each step depends on prior actions, and errors in ordering, sequencing, or overuse of tests carry both clinical and economic consequences. Similarly, tumor board decision-making requires integrating pathology, CT and MRI findings, clinical guidelines, comorbidities, and trial eligibility into a coordinated treatment plan across specialties. In these settings, correctness depends on the pathway as well as the endpoint. A correct diagnosis reached through excessive or misordered interventions is not clinically equivalent to an efficient, guideline-concordant workup.

A process-aware benchmark for medical AI agents should assess the entire decision trajectory (see Fig 1B). Three dimensions are particularly important. Action appropriateness captures whether the system selects clinically sensible actions at the right point in the workflow. Process safety captures whether it avoids unsafe steps, such as invasive investigations without indication, omitted red-flag screening, or treatment recommendations made without checking contraindications. Resource stewardship captures whether it avoids unnecessary tests, procedures, referrals, or costs.

Because clinical trajectories rarely have a single ground truth, future benchmarks should define acceptable ranges of practice rather than one fixed answer. Reference trajectories should be derived from expert consensus, clinical guidelines, simulated patient encounters, and, where appropriate, observed care pathways. Multidisciplinary clinical panels and formal consensus methods could help bound acceptable variation while allowing benchmarks to be updated as evidence and practice evolve. Such benchmarks should capture the full structure of clinical decision-making, including the sequence of clinical findings, laboratory and imaging requests, referrals, treatment decisions, and safety-critical omissions.

Building these benchmarks will be technically and financially demanding, requiring robust simulated clinical environments and sustained expert input. Their design must reflect the intended clinical role of the agent. In the foreseeable future, medical AI agents are likely to act primarily in clinician-supporting roles rather than replacing them. In tumor board settings, agents may be most useful when they broaden multidisciplinary review by identifying relevant trials, off-label treatments and additional evidence. More autonomous systems that execute clinical workflows would require stricter evaluation. Benchmark metrics must also be designed carefully to avoid reward hacking, where agents optimize benchmark scores without improving clinical utility. The goal is to permit legitimate variation in clinical practice while identifying trajectories that are unsafe, inefficient, or poorly justified. As medical AI moves from prediction to action, benchmarks must evolve from scoring isolated answers to evaluating clinically plausible decision trajectories.

## Abbreviations

AI: artificial intelligence
LLM: large language model
